# The influence of cytokine gene polymorphisms on the risk of developing gastric cancer in patients with Helicobacter pylori infection

**DOI:** 10.2478/raon-2014-0041

**Published:** 2015-08-21

**Authors:** David Stubljar, Samo Jeverica, Tomislav Jukic, Miha Skvarc, Tadeja Pintar, Bojan Tepes, Rajko Kavalar, Borut Stabuc, Borut Peterlin, Alojz Ihan

**Affiliations:** 1 Institute of Microbiology and Immunology, Ljubljana, Slovenia; 2 Medical faculty of Osijek, Osijek, Croatia; 3 Department of Abdominal Surgery, University Clinical Centre Ljubljana, Ljubljana, Slovenia; 4 Abacus Medico Diagnostic Centre Rogaska, Rogaska Slatina, Slovenia; 5 Department of Pathology, University medical Centre Maribor, Maribor, Slovenia; 6 Department of Gastroenterology, University Medical Centre Ljubljana, Ljubljana, Slovenia; 7 Clinical Institute of Medical Genetics, University Clinical Centre Ljubljana, Ljubljana, Slovenia

**Keywords:** *Helicobacter pylori*, gene polymorphisms, gastric cancer, chronic gastritis

## Abstract

**Background:**

*Helicobacter pylori* infection is the main cause of gastric cancer. The disease progression is influenced by the host inflammatory responses, and cytokine single nucleotide polymorphisms (SNPs) may have a role in the course of the disease. The aim of our study was to investigate proinflammatory cytokine polymorphisms, previously associated with the development of gastric cancer, in a Slovenian population.

**Patients and methods.:**

In total 318 patients and controls were selected for the study and divided into three groups: (i) patients with gastric cancer (n = 58), (ii) patients with chronic gastritis (n = 60) and (iii) healthy control group (n = 200). *H. pylori* infection in patient groups was determined by serology, histology and culture. Four proinflammatory gene polymorphisms were determined (IL-1β, IL-1ra, TNF-α, TLR-4) in all subjects.

**Results:**

We found a statistically significant difference between males and females for the groups (p = 0.025). Odds ratio (OR) for gastric cancer risk for females was 0.557 (95% confidence interval [CI]: 0.233–1.329) and for chronic gastritis 2.073 (95% CI: 1.005–4.277). *IL-1B-*511*T/T homozygous allele for cancer group had OR = 2.349 (95% CI: 0.583–9.462), heterozygous *IL-1B-*511*T had OR = 1.470 (95% CI: 0.583–3.709) and heterozygotes in *TNF-A*-308 genotype for chronic gastritis had OR = 1.402 (95% CI: 0.626–3.139). Other alleles had OR less than 1.

**Conclusions:**

We could not prove association between gastric cancer and chronic gastritis due to *H. pylori* in any cytokine SNPs studied in Slovenian population. Other SNPs might be responsible besides infection with *H. pylori* for the progression from atrophy to neoplastic transformation.

## Introduction

Gastric cancer is the fourth most commonly diagnosed cancer and the second most common cause of cancer-related death worldwide.^1^ The incidence of gastric cancer in Slovenia is among the highest in Europe with the crude incidence rate in male population of 28.6/100 000.^2^–^4^ Gastric cancer is multifactorial disease. Environmental and host genetic factors influence the development of gastric cancer. The most important is *Helicobacter pylori* (*H. pylori*) infection. It is belived that roughly 65–80% of gastric cancers are associated with *H. pylori* infection. However, only a minority (1–2%) of infected individuals will develop gastric cancer during their lifetime.^5^,^6^ Gastric carcinogenesis is a multistep process that starts with chronic active gastritis and continues through the development of gastric atrophy, and metaplasia, to reach gastric cancer stage at the end of that process, lasting tipically between 30–50 years.^7^–^10^ In addition to *H. pylori* infection, several host genetic factors are important for the development of gastric cancer, especially several single nucleotide polymorphisms (SNPs) and/or point mutations in genes that affect gastric acid secretion and innate immune response to infection.^11^–^14^ Polymorphisms in cytokine genes may influence the level of the cytokine production by the host, and consequently influence the disease outcome.^15^ The immune response to *H. pylori* is important for the development of gastric cancer due to the recognition of pathogenic elements and induced synthesis and secretion of inflammatory cytokines, resulting inflammation, what can lead to severe gastric immunopathology and cancer.^16^

Interleukin 1b (IL-1b) is the main cytokine secreted in response to *H. pylori* infection. It has a strong pro-inflamatory activity and inhibits gastric acid secretion. IL-1b is 100-times more potent inhibitor of acid secretion than proton pump inhibitors.^17^ Inhibition of acid secretion may lead to the spread of bacteria from the antrum to the corpus, and consequently the development of corpus predominant gastritis which further leads to the development of gastric cancer.^18^,^19^ Three polimorphisms were described in the *IL-1B* gene at positions −31, −511 and +3954 from the transcription start site.^18^,^20^ *IL-1B*-31*C and *IL-1B*-511*T alleles are associated with decreased acidity in the stomach (hypochlohydria) in response to the infection with *H. pylori*.^18^ IL-1b receptor antagonist (IL-1ra) polymorphisms have also been associated with the level of IL-1b secretion. Genotype IL-1RN*2 is associated with higher secretion of IL-1b, most probably through the reduction of its receptor antagonist IL-1ra.^20^,^21^

Tumor necrosis factor-a (TNF-*α*) is a central mediator of the immune response. Several polymorphisms are known in the promoter region of *TNF-A* gene of which −308*G>A was associated with increased production of TNF-α in response to the infection, and increased gastric cancer risk.^22^–^24^ El-Omar *et al*.^25^ and Machado *et al*.^26^ found that subject with this polymorphism have almost two-fold increased risk of gastric cancer.

Recently, a functional polymorphism at the position +896, in exon 4 of the *Toll-like receptor-4* (*TLR-4)* gene, has been described. This A>G transition results in an alteration of the extracellular domain of TLR-4, that causes hyporesponsiveness to LPS, reduced epithelial TLR-4 density and exaggerated inflammatory cytokine response.^27^ A recent study has reported an association of *TLR-4* gene polymorphisms with gastroduodenal diseases such as gastric atrophy and hypochlorhydria.^28^,^29^ *TLR-4* substitution was associated with noncardia gastric cancer.^30^,^31^

The aim of our study was to determine the prevalence of the selected pro-inflammatory cytokine polymorphisms in the Slovenian population of patients with gastric cancer and chronic gastritis, and compare its prevalence with the prevalence in the normal healthy population, to see if high incidence of gastric cancer in Slovenian population could be, at least partially, attributed to the higher prevalence of those proinflammatory polymorphisms in the genes for IL-1β, IL-1ra, TNF-α and TLR-4.

## Patients and methods

### Patients

In total 318 patients and controls were included in the study devided into three groups: (i) consecutive patients with gastric cancer (n = 58), (ii) consecutive patients with chronic gastritis due to *H. pylori* (n = 60) and (iii) healthy control group (n = 200). Study was conducted as a case-control study, where the cancer patients represented one group and the gastritis patients represented the other group. Subjects for the healthy control group were randomly selected from the pool of representative blood samples of Slovenian healthy adults, to be matched for age and sex. All subjects were informed about the inclusion in the study and agreed to it in writing form. National medical ethics committee reviewed and cleared the protocol of the study.

### Histopathology, serology and culture

Patients in the gastric cancer group had the histological type of cancer determined using the Lauren’s classification that differentiates among intestinal, diffuse and mixed or indetermined type adenocarcinoma. In the group of patients with chronic gastritis two biopsies were obtained from corpus and antrum, and the histological diagnosis was determined in accordance with the Huston modification of Sydney classification for gastritis.^7^,^32^

Serological confirmation of *H. pylori* infection in the gastric cancer group was confirmed by the quantitative IgG ELISA test GAP®-IgG (Biomerica, USA) from human serum. Test was performed in accordance to instructions by the manufacturer.^33^

*H. pylori* culture was performed in the gastritis group from two biopsy samples of antrum and corpus, respectively. Biopsy samples were transported to the laboratory in Portagerm pylori transport medium (Biomerieux, France). In the laboratory, samples were homogenized in 1 mL of phosphate buffer (PBS) and 0.5 mL of the homogenate was inoculated onto two selective agar plates: Pylori agar (Biomerieux, France) and Brucella agar supplemented with human blood and antibiotic mixture (BBL, USA). Culture media were incubated at 37 °C for 72 hours in microaerophilic conditions. The identification of typical colonies was confirmed using Gram stain and the proof of enzymes: urease, catalase and oxidase.

### Genotyping

Genomic DNA was extracted from the whole blood samples with EDTA using automated system for DNA isolation Magna Pure Compact Nucleic Acid Isolation Kit I (Roche Applied Science, Germany) on fully automated platform MagNa Pure Compact System in accordance to the instructions by the manufacturer.^34^ Complete nucleotide sequences of individual genes for inflammatory cytokine *IL-1β* (rs16944), *TNF-α* (rs1800629) and *TLR-4* (rs4986790) were looked into online databases National Center for Biotechnology Information (NCBI; www.ncbi.nlm.nih.gov) and ENSEMBLE (www.ensembl.org). The sequences were examined with the help of the software package Vector NTI Advance 11 (Invitrogen, Carlsbad, CA, USA).^21^,^25^,^26^ Polymorphisms genotyping was performed using the KASP technology (KBioscience competitive Allele-Specific PCR) using primers and reagents Kasp On Demand (KOD) (KBioscience, UK). 120 bp long reference sequences were sent to the manufacturer, upon which the appropriate primers and probes were designed (Table 1).

The amplification of genomic DNA and the detection of polymorphisms were performed using the real-time polymerase chain reaction (PCR) apparatus LightCycler 480II (Roche Diagnostics GmbH, Germany). A touchdown protocol provided by the manufacturer was used: 94 °C for 15 min; 10 cycles of 94 °C for 10 s, 61 °C for 60 s (the anniling temperature dropped 0.6 °C per cycle to reach the annealing temperature of 55 °C) then; 26 cycles of 94 °C for 10 s, 55 °C for 60 s. *IL-1RN* gene contains a variable number of 86 base pair long tandem repeats (VNTR).^19^ Genomic DNA was amplified and PCR products were separated by the 1.5% agarose gel electrophoresis. Primers to detect IL-1RN*2/2 (TIB Molbiol, Germany) were used. We have used forward primer: 5′-CCCCTCAGCAACACTCC-3′, reverse primer: 5′-GGTCAGAAGGGCAGAGA-3′. Cycling conditions for the PCR were 95 °C for 15 min; 30 cycles of 94 °C for 30 s and 61 °C for 30 s; 72 °C for 60 s and 15 min at 72 °C. PCR reaction with the final volume of 25 μl was used, containing 12.5 μl of twice the reaction mixture of HotStartTaq Plus, 0.75 μl of each primer with a concentration of 10 μM, 8.5 μl of ddH_2_O and 2.5 μl of sample DNA.

There are 5 versions of alleles. Allele 1, 2, 3, 4 and 5 carries 4, 2, 5, 3 and 6 repeats, respectively.^20^,^35^ Due to easier statistical analysis the allele polymorphisms were divided into short and long, the short allele being allele 2 and the long allele being those with 3 repeats or more (alleles 1, 3, 4, and 5).^26^

### Statistical analysis

The SPSS Statistics 21 (IBM, USA) software package was used for the statistical analysis. The Hardy-Weinberg equilibrium (HWE) of alleles in each individual locus was assessed. The degrees of freedom for HWE were calculated as the number of genotypes subtracted with the number of alleles. If the value of the c^2^ was less than 3.84, the frequencies of the population were in HWE. For all genotypes, the homozygote of the common allele was used as the reference. The *IL-1B*, *IL-1RN*, *TNF-A* and *TLR-4* genotype frequencies for each polymorphism were compared by 2-sided Pearson c^2^ test, to evaluate the genotype distributions of categorical variables between each group of cases and controls, and to see if there was any association between the tested variables. The odds ratios (ORs) and the 95% confidence interval (95% CI) were assessed using logistic regression analysis with the reference category being healthy controls. ORs for different groups were adjusted for sex only. Statistical differences were considered to be significant at a *P* value < 0.05.

## Results

Patients with diagnosed chronic gastritis due to *H. pylori* and gastric cancer were investigated compared to healthy controls. The average age of individuals and gender ratio were comparable in all groups (Table 2). We included 198 subjects and controls in the study meeting the necessary initial criteria: 108 healthy control subjects with no underlying conditions, 32 patients with intestinal type of gastric adenocarcinoma and 58 patients with chronic gastritis and positive *H. pylori* infection were included and processed for statistical analysis.

The genotype frequencies distribution among cytokine polymorphisms are presented in Table 3. Comparison of genotype frequencies between intestinal adenocarcinoma group and atrophic gastritis group and healthy controls showed no significant difference (p > 0.05). P-value of 0.084 for IL-1β showed closest statistical difference between the diagnosis severe progression and influence of genetic polymorphisms. However, there was a statistically significant difference between males and females compared between the groups (p = 0.025) (Table 3). The sex-adjusted OR of gastric cancer among *H. pylori* positive subjects was 0.557 (95% CI: 0.233–1.329; p = 0.187) and of chronic gastritis 2.073 (95% CI: 1.005–4.277; p = 0.048). Males were taken as reference.

In the gastric carcinoma patients, *IL-1B-*511*T/T homozygous allele represented 15.6% (5/32) of the case subjects, which was proportionally higher than in control group (12.0%; 13/108), however statistically with an OR of 2.349 (95% CI: 0.583–9.462) was not confirmed. Carriers of heterozygous *IL-1B-*511*T allele in cancer group (53.1%, 17/32) also showed no difference against control group (49.1%, 53/108) despite the OR = 1.470 (95% CI: 0.583–3.709). For atrophic gastritis group there was no statistically difference compared to control group (Table 4). Carriers of the proinflammatory *IL-1B*-511*T allele (both *IL-1B*-511T homozygotes and *IL-1B*-511 heterozygotes) had also no increased risk for gastric cancer (OR = 1.570; 95% CI: 0.644–3.825) or chronic gastritis (OR = 0.480; 95% CI: 0.232–0.996). The associated OR value was even smaller than for homozygotes alone with low frequency of homozygous controls (Table 4). According to Pearson’s χ^2^ frequency distribution of *IL-1B*-511*T carriers were statistically significant in combination for specific diagnose (p = 0.021; F = 7.760) (data not shown).

The observed associations between *IL-1RN* VNTR genotype carriers (IL-1RN*L/2) and the risk of gastric carcinoma or atrophic gastritis had meaningless OR = 1.064 (95% CI: 0.436–2.597), OR = 1.052 (95% CI: 0.473–2.341), respectively. Furthermore short allele had no statistical association with developing the disease.

In a logistic regression model that included the other genetic markers (*TNF-A* and *TLR-4*), there were no statistical significant differences adjusted to control group and common alleles. Heterozygotes in *TNF-A*-308 genotype had also no statistically significant excess for the chronic gastritis (OR = 1.402; 95% CI: 0.626–3.139) (Table 3). *TNFA*-308*A carriers (both *TNF-A*-308*A homozygotes and *TNF-A*-308 heterozygotes) had even less probability with an OR of 1.217 (95% CI: 0.556–2.667) for gastritis (Table 5).

Pearson correlation model for all *IL-1B-*511, *IL-1RN* VNTR, *TNF-A*-308 and *TLR-4*+896 genotypes was performed and showed no statistical significance between them (p > 0.01). However correlation between *IL-1B* and *IL-1RN* was found (Pearson’s R = 0.300; p < 0.001). Furthermore, there was no evidence of the association between the 55 (28.9%) carriers of *IL-1B-*511*T and IL-1RN*2 alleles (OR = 1.489; 95% CI: 0.660–3.361) for the risk of gastric cancer (data not shown). There was also no association for chronic gastritis. Moreover, combined T and 2 allele carriers had even lesser risk associated with developing gastric cancer than each allele separately.

## Discussion

This is the first study on Slovenian population that checked variants or polymorphisms in genes responsible for cytokine secretion that may contribute to the different outcomes of infection and the development of gastric lesions. Our results showed that there was a statistical difference between genders on the outcome of infection with *H. pylori* (p = 0.025). Males were more predominant to develop gastric cancer than females (female OR = 0.557). Meanwhile females had 2-fold greater probability to develop chronic gastritis (OR = 2.073; 95% CI: 1.005–4.277). Our results were consistent with reported results in studies stated by Chandanos and Lagergren^4^, and Dixon *et al*.^32^ All investigated polymorphism unfortunately showed no associations with disease prediction.

*IL-1B* polymorphisms were not statistically associated with the prediction of each diagnose as according, however p-value to determine association between polymorphism and outcome of infection (diagnose severity: gastritis or cancer) was 0.084. Frequency distribution in our population showed that *IL-1B*-511*C homozygote allele was most frequent in chronic gastritis group (58.8%). According to our knowledge such results were not found in any other study. Genotype frequencies for cancer group were coincided with control group. Studies in Caucasian and Asian populations have shown that polymorphisms in the genes *IL-1B* and *IL-1RN* were in conjunction with an increased risk for hypochlohydria and gastric carcinoma.^13^ According to our findings, individuals carrying the *IL-*1B-511*T/T allele compared to control group showed an increased OR for gastric cancer. Heterozygotes for *IL-1B* gene (*IL-1B*-511*T carriers) and both homozygotes and heterozygotes for T allele, also showed increased OR for developing gastric cancer. Although the OR values were evaluated it would be exaggerated to affirm that these polymorphisms could indicate on the risk for developing intestinal adenocarcinoma, because the power of our statistical analysis was really poor with p-values less then 0.05 and wider 95% CI. However allele combination (T/T and C/T) showed statistically significant association with diagnose prediction (p = 0.021). Percent of *IL-1B*-511*T carriers in cancer group has reached almost 69% of tested individuals.

El-Omar *et al*.^24^ have identified the inflammatory profile of genetic polymorphisms in the genes for IL-1β (*IL-1B* -511*T) and IL-1ra (IL-1RN*2/2) to increase the risk of developing gastric cancer.^17^ In our population there was 40.6% of short allele carriers diagnosed with cancer but no statistical difference to predict the disease was observed (Table 4). The correlated association between *IL-1B* and *IL-1RN* proinflammatory genotypes (*IL-1B*-511*T carriers and IL-1RN*2 homozygotes) and risk for gastric cancer was also determined (p < 0.001 and Pearson’s R = 0.300). These results indicated that IL-1RN*2/2 gene is recessive in combination with T carriers in *IL-1B*.^36^ The genotype frequencies for individuals with gastric cancer or even chronic gastritis were even smaller than in control group. Results should be taken caustiously because in our population only 2% of cancer patients or patients with gastritis and 12% of controls had IL-1RN*2/2.

The present study has showed that *TLR-4* polymorphism is not associated with the development of the premalignant gastric abnormalities of hypochlorhydria and atrophy, or with increased risk of gastric adenocarcinoma. No association was seen with cancer although this polymorphism has been associated with risk of other inflammatory conditions. The polymorphism was associated with hyporesponsiveness to bacterial LPS.^37^ The association of the *TLR-4*+896A>G polymorphism identifies subjects who have an increased risk of severe inflammation and subsequently, development of hypochlorhydria and gastric atrophy, which are regarded as the most important precancerous abnormalities.^27^ However, our results were comparable to those by Garza-Gonzales^28^ that the *TLR-4* polymorphism did not play a role in the development of gastric premalignancies.

*H. pylori* infection also enhances the mucosal production of TNF-α. TNF-α is not as potent inhibitor of gastric acid secretion as IL-1β.^38^ Although El-Omar *et al*.^20^ and Machado *et al*.^26^ found an almost two-fold increase in risk for gastric cancer, several studies have not found an association between *TNF-A*-308*A and gastric cancer risk.^39^–^41^ The *TNF-A*-308*A allele has been found in association with an increased risk of *cagA* positive infections and gastric cancer by Zambon *et al*.^23^ and Yea *et al*.^42^ also found no significant association between the *TNF-A*-308 polymorphism and the severity of gastric disease (carcinoma, gastritis, gastric ulcers, duodenal ulcers). However our results have not confirmed that and were coincided with results of Tseng *et al*.^43^, who investigated polymorphisms in Jamaican children. Meanwhile the G allele has been found to be associated with peptic ulcer, which commonly accompanies gastritis^32^, and concomitant *H. pylori* infection, compared to those without ulcerations.^44^ Mucosal expression levels of TNF-α was lower in *H. pylori*-infected individuals with duodenal ulcers. Heterozygous G carriers in our population were slightly drawn near with development of chronic gastritis (OR = 1.402; 95% CI: 0,626–3,139), but again the p-value was 0.411 and the association was not confirmed.

The reduced number of samples available for statistical analysis may have harmed our results. We have found no indications that the infection with *H. pylori* in a given inflammatory genotype of could result in an inflammatory response, and then gastritis or cancer. We have also showed that the presence of *IL-1B*-511 genotype for the inflammatory cytokine was inclined to the difference between intestinal type of gastric cancer, chronic gastritis and healthy controls. However statistically it was not associated entirely and could not be used to identify people at increased risk. On the other hand, cytokine gene polymorphisms represent just one component of complex interactions among host, pathogen, and environmental factors involved in gastric carcinogenesis, what was definitly confirmed with statistical difference between genders. Only combination of *H. pylori* and host-associated risk factors do not always allow evaluation of gastric carcinoma risk. The progression from atrophy to neoplastic transformation depends on other factors, including diet and different pathogenesis of *H. pylori* strains.^5^,^7^ Ando *et al.*^45^ have found that patients who develop duodenal ulcer disease are protected from gastric cancer. Both conditions are associated with *H. pylori*, but duodenal ulcers are associated with an antrum predominant gastritis, low prevalence of gastric atrophy, and very high acid secretion. On the contrary, gastric cancer patients develop corpus predominant gastritis, multifocal atrophic gastritis, and hypochlorhydria. Proinflammatory genotypes of the *IL-1B* gene, through its induction of gastric atrophy and gastric acid inhibition, increase the risk of gastric atrophy.

The number of cases in our study was small. In the study, in cancer group, we only included patients with intestinal type of gastric cancer, however in gastritis group we included all gastritis types, not only those with accompanied atrophy. Individuals with extensive corpus gastritis develop hypochlorhydria and gastric atrophy, which are presumptive precursors of gastric cancer.^20^ Another drawback is that we have not determined bacterial strain (*vac A*, *cag A*) as it was done by Figueiredo *et al.*^46^ and Zambon *et al.*^23^ Anyway, now we have learnt that the assessment of patients with *H. pylori* infection and its strain is very important and concluded that eradication of bacteria has essential meaning. We recommend that not only screening for *H. pylori* also the strain determination should have some diagnostic value, especially in the patients who already developed gastritis. Furthermore, for such patients assessment of disease progression (atrophic or metaplastic gastritis) could be followed by polymorphism determination. The statistical power of our pilot study was very poor and we could not evaluate it to the whole Slovenian population, but for further polymorphism investigations it is necessary to include more patients with different disease progression. Our study design was considered good, because our study population was not heterogenic. Untill now we cannot predict the disease based only on single polymorphism.

## Conclusions

Altogether, our findings indicated that host genotype as well as *H. pylori* infection could be important for greater risk for developing gastric cancer. However, those parameters alone could not predict the incidence of the disease. For more accurate analysis of the impact of genetic polymorphisms and identification of people with an increased risk for developing the disease, it would be necessary to expand the study and include a larger number of subjects, especially patients with gastric cancer.

## Figures and Tables

**TABLE 1.** Primers and probes sequences used in the KASP assays

**Gene**	**Probes**	**Primers**
**IL-1β −511 C/T**	**FAM**	5′-GCCCCAGCCAAGAAAGGTCAATTTT-3′
	5′-GGGTGCTGTTCTCTGCCTC**G**-3′	
	**VIC**	
	5′-GGGTGCTGTTCTCTGCCTC**A**-3′	
**TNF-α −308 G/A**	**FAM**	5′-GAGGCAATAGGTTTTGAGGGGCAT-3′
	5′-GGAGGCTGAACCCCGTCC**T**-3′	
	**VIC**	
	5′-GAGGCTGAACCCCGTCC**C**-3′	
**TLR-4 +896 A/G**	**FAM**	5′-CACTCACCAGGGAAAATGAAGAAACATT-3′
	5′-GCATACTTAGACTACTACCTCGATG**A**-3′	
	**VIC**	
	5′-CATACTTAGACTACTACCTCGATG**G**-3′	

**TABLE 2.** Demographic profiles of subjects

**Demographic profile**	**Gastric cancer** (n = 58)		**Gastritis** (n = 60)		**Controls**
		
	Intestinal	Diffuse	Atrophic	Metaplastic	/
**No. of subjects**	32 (55%)	26 (45%)	51 (85%)	9 (15%)	200
**Age (years)**	52 ± 10	52 ± 10	52 ± 10	58 ± 13	49 ± 5
**Gender**					
Male	22 (38%)	18 (31%)	20 (33%)	2 (3%)	100 (50%)
Female	10 (17%)	8 (14%)	31 (52%)	7 (12%)	100 (50%)

**TABLE 3.** Pearson’s χ^2^ analysis for association between frequencies of cytokine polymorphisms and patients with intestinal type gastric adenocarcinoma, atrophic chronic gastritis and healthy controls

	**Intestinal adenocarcinoma**		**Atrophic gastritis**		**Controls**		**Pearson’s χ^2^**	**p-value**
		
	(n = 32)		(n = 51)		(n = 108)			
***IL-1B* −511**							8.214	0.084
C/C	10	31.3%	30	58.8%	42	38.9%		
C/T	17	53.1%	18	35.3%	53	49.1%		
T/T	5	15.6%	3	5.9%	13	12.0%		
***IL-1RN***							4.377	0.357
L/L	17	53.1%	33	64.7%	63	58.3%		
L/2	13	40.6%	15	29.4%	32	29.6%		
2/2	2	6.3%	2	3.9%	13	12.0%		
***TNF-A −308***							4.796	0.309
G/G	27	84.4%	36	70.6%	83	76.9%		
G/A	5	15.6%	15	29.4%	22	20.4%		
A/A	0	0.0%	0	0.0%	3	2.8%		
***TLR-4 +896***							3.355	0.500
A/A	30	93.8%	46	90.2%	90	83.3%		
A/G	2	6.3%	5	9.8%	17	15.7%		
G/G	0	0.0%	0	0.0%	1	0.9%		
**Gender**							7.355	0.025
M	22	68.8%	20	39.2%	60	55.6%		
F	10	31.3%	31	60.8%	48	44.4%		

F = female; M = male

**TABLE 4.** Genotype polymorphisms odds ratios (ORs) and 95% confidence intervals (CIs) for gastric cancer and atrophic gastritis subjects

	**Intestinal adenocarcinoma** (n = 32)	**OR**	**95% CI**	**p-value**	**Atrophic gastritis** (n = 51)	**OR**	**95% CI**	**p-value**
***IL-1B* −511**								
C/C	10 (31.3)	reference			30 (58.8)	reference		
C/T	17 (53.1)	1.470	0.583–3.709	0.414	18 (35.3)	0.489	0.228–1.050	0.067
T/T	5 (15.6)	2.349	0.583–9.462	0.230	3 (5.9)	0.416	0.099–1.757	0.233
***IL-1RN***								
L/L	17 (53.1)	reference			33 (64.7)	reference		
L/2	13 (40.6)	1.064	0.436–2.597	0.891	15 (29.4)	1.052	0.473–2.341	0.900
2/2	2 (6.3)	0.394	0.072–2.162	0.394	2 (3.9)	0.400	0.081–1.988	0.263
***TNF-A −308***								
G/G	27 (84.4)	reference			36 (70.6)	reference		
G/A	5 (15.6)	0.704	0.236–2.099	0.528	15 (29.4)%	1.402	0.626–3.139	0.411
A/A	0 (0.0)	0	0	0	0 (0.0)	0	0	0
***TLR-4 +896***								
A/A	30 (93.8)	reference			46 (90.2)	reference		
A/G	2 (6.3)	0.326	0.066–1.603	0.168	5 (9.8)	0.499	0.149–1.668	0.259
G/G	0 (0.0)	0	0	0	0 (0.0)	0	0	0
**Gender**								
M	22 (68.8)	reference			20 (39.2)	reference		
F	10 (31.3)	0.557	0.233–1.329	0.187	31 (60.8)	2.073	1.005–4.277	0.048

Reference category for groups was set to control group. Referent allele was common homozygote; F = female; M = male

**TABLE 5.** Frequencies of genotype carriers, odds ratios (ORs) and 95% confidence intervals (CIs) for gastric cancer and atrophic gastritis subjects

	**Intestinal adenocarcinoma** (n = 32)	OR	95% CI	p-value	**Atrophic gastritis** (n = 51)	OR	95% CI	p-value
***IL-1B* −511**								
C/C	10 (31.3)	reference			30 (58.8)	reference		
T carrier	22 (68.7)	1.570	0.644–3.825	0.321	21 (41.2)	0.480	0.232–0.996	0.049
***IL-1RN***								
L/L	17 (53.1)	reference			33 (64.7)	reference		
2 carrier	15 (46.9)	0.947	0.408–2.200	0.900	17 (33.3)	0.905	0.429–1.912	0.794
***TNF-A −308***								
G/G	27 (84.4)	reference			36 (70.6)	reference		
A carrier	5 (15.6)	0.590	0.201–1.730	0.336	15 (29.4)	1.217	0.556–2.667	0.623
***TLR-4 +896***								
A/A	30 (93.8)	reference			46 (90.2)	reference		
G carrier	2 (6.3)	0.318	0.068–1.487	0.145	5 (9.8)	0.435	0.135–1.407	0.165
**Gender**								
M	22 (68.8)	reference			20 (39.2)	reference		
F	10 (31.3)	0.561	0.237–1.329	0.189	31 (60.8)	2.068	1.015–4.213	0.045

Reference category for groups was set to control group. Referent allele was common homozygote; F = female; M = male
